# A novel fixation method for unstable ankle fractures in the elderly using dual percutaneous tibiotalar cannulated screws

**DOI:** 10.1093/jscr/rjab311

**Published:** 2021-07-24

**Authors:** Daniel W Hancock, Louis Hainsworth, Alanna K Pentlow

**Affiliations:** Department of Trauma & Orthopaedics, Weston General Hospital, Grange Road, Uphill, Weston-Super-Mare, Somerset BS23 4TQ, UK; Department of Trauma & Orthopaedics, Weston General Hospital, Grange Road, Uphill, Weston-Super-Mare, Somerset BS23 4TQ, UK; Department of Trauma & Orthopaedics, Weston General Hospital, Grange Road, Uphill, Weston-Super-Mare, Somerset BS23 4TQ, UK

## Abstract

Ankle fractures in the elderly are often complicated by osteoporosis and poor skin quality, resulting in poorer outcomes. This retrospective case-series describes a novel minimally invasive fixation method for managing unstable ankle fractures in the high-risk elderly patient. Six elderly patients who underwent dual tibiotalar cannulated screw fixation for unstable ankle fractures between January 2019 and 2020 were identified. Data on post-operative pain scores, mobility and complications were collected. Pre- and post-operative radiographs were analysed for fracture type and complications. Functional outcomes were excellent with 83.3% of patients pain free, and 100% ambulatory with walking-aids at 10.25 months follow-up. Four of the six patients (66.7%) had satisfactory post-operative radiographs, and there were no soft tissue injury or infections due to the surgery. Dual percutaneous tibiotalar cannulated screws can be used to manage unstable ankle fractures in the low demand elderly patient, resulting in excellent functional outcomes.

## INTRODUCTION

Ankle fractures are a commonly occurring injury making up a quarter of all lower limb fractures, with an incidence as high as 184 per 100 000 persons per year [1]. With an ageing population the prevalence of these injuries is rising, with ~30% of all ankle fractures occurring in the elderly [1].

Ankle fractures in the elderly are a challenging injury to manage. These patients are frequently co-morbid with poor-quality tissues and osteoporotic bone [2]. These issues have significant implications for both non-operative and operative management. Several studies have sought to identify the optimal management of ankle fractures with the recent AIM (Ankle Injury Management) trial concluding that treatment of ankle fractures in older adults should focus on obtaining and maintaining reduction until union by the most conservative means possible, with good results from close contact casting [3]. In a younger patient population, conservative management with the use of non-weight bearing is tolerated, whereas this is a much more significant challenge in an elderly patient with poor functional status.

Considering operative management, the co-morbid elderly patient not only has significant risks from the anaesthetic but also the procedure itself [2]. Osteoporotic bone leads to challenges in maintaining fixation to allow union and weight-bearing [4]. Alongside this, diabetes and poor-quality tissues lead to significant risks of wound healing problems with infection and breakdown occurring frequently [2, 5].

The current concepts with ankle fracture fixation have increasingly sought to use less invasive techniques to reduce and maintain the fracture while allowing weight bearing. Hindfoot nails have been used allowing the elderly patient to return to full weight bearing immediately [6]. However, this traverses both the ankle and subtalar joints and have their own considerable issues, including pain, failure and significantly reduced mobility [1].

We describe our experience and early follow-up using a novel minimally invasive operative technique for managing unstable ankle fractures in co-morbid elderly patients.

## MATERIALS AND METHODS

The trauma database at a district general hospital was reviewed for all patients who underwent fixation of unstable ankle fractures via dual percutaneous tibiotalar cannulated screws between January 2018 and January 2020. Six patients were identified and included in the retrospective case-series, with operations occurring between 8 January 2019 and 16 January 2020. Demographic data were collected for each patient including age, gender, ASA (American Society of Anaesthesiologists) grade, smoking status and co-morbidities.

Preoperative ankle fracture radiographs were analysed retrospectively and classified as per the number of malleoli involved and by the Danis–Weber classification. Four (66.7%) tri-malleolar fractures and two (33.3%) bimalleolar fractures were included. Using the Danis–Weber classification, there were three (50%) Weber-B, two (33.3%) Weber-C and one fracture without lateral malleolus involvement.

Each patient underwent manipulation under anaesthesia of the ankle, and then percutaneous fixation of the tibia talar joint with two large cannulated screws, using the same technique as used for ankle fusion surgery. Five patients received two 6.5-mm cannulated screws, and one patient received two 8.0-mm cannulated screws. Operative times were recorded retrospectively from the electronic database.

Patients were then placed in a plaster cast and allowed to fully weight-bear. The aim was to hold the talus beneath the tibia whilst the fractures united.

Follow-up duration was between two and 18 months (mean follow-up of 10.25 months). Each patient was evaluated for post-op pain using a numeric rating score and mobility was compared to preoperative status.

Post-operative radiograph follow-up duration was between 6 and 20 weeks (mean follow-up of 9 weeks). They were evaluated for any evidence of talar shift, widening of syndesmosis, non or mal-union, screw loosening or periprosthetic fractures. In addition, we noted any removal of screws and further complications relating to screw placement, such as ulceration.

## RESULTS

All six patients (100%) were female, with a mean age of 86.83 years (Range of 70–99). Half had type two diabetes, and there were no active smokers. Each patient was significantly co-morbid with an average ASA grade of three. Two patients (33.3%) had established ulceration over the ankle preoperatively. The average operative time was 29 min with a range of 23–44 min.

### Post-operative radiograph review

**Figure 1:**
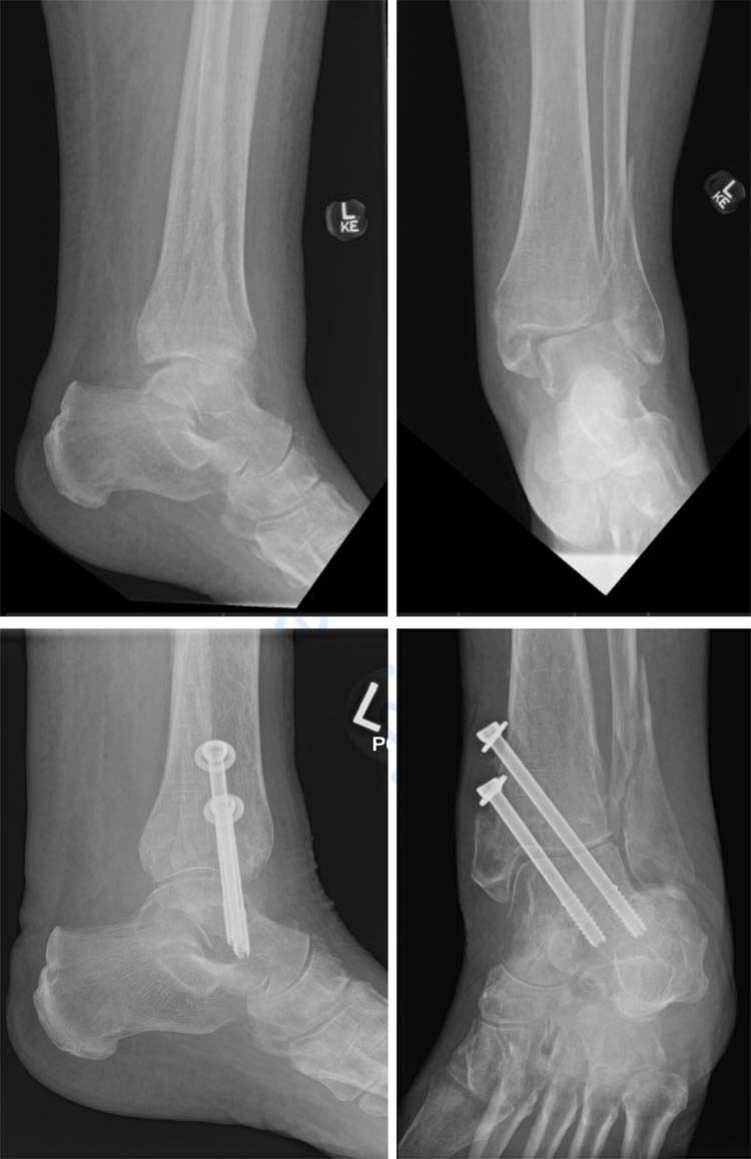
Top—Bimalleolar ankle fracture with talar shift. Bottom—Radiographs at 3 months follow-up showing maintained tibiotalar alignment.

**Figure 2:**
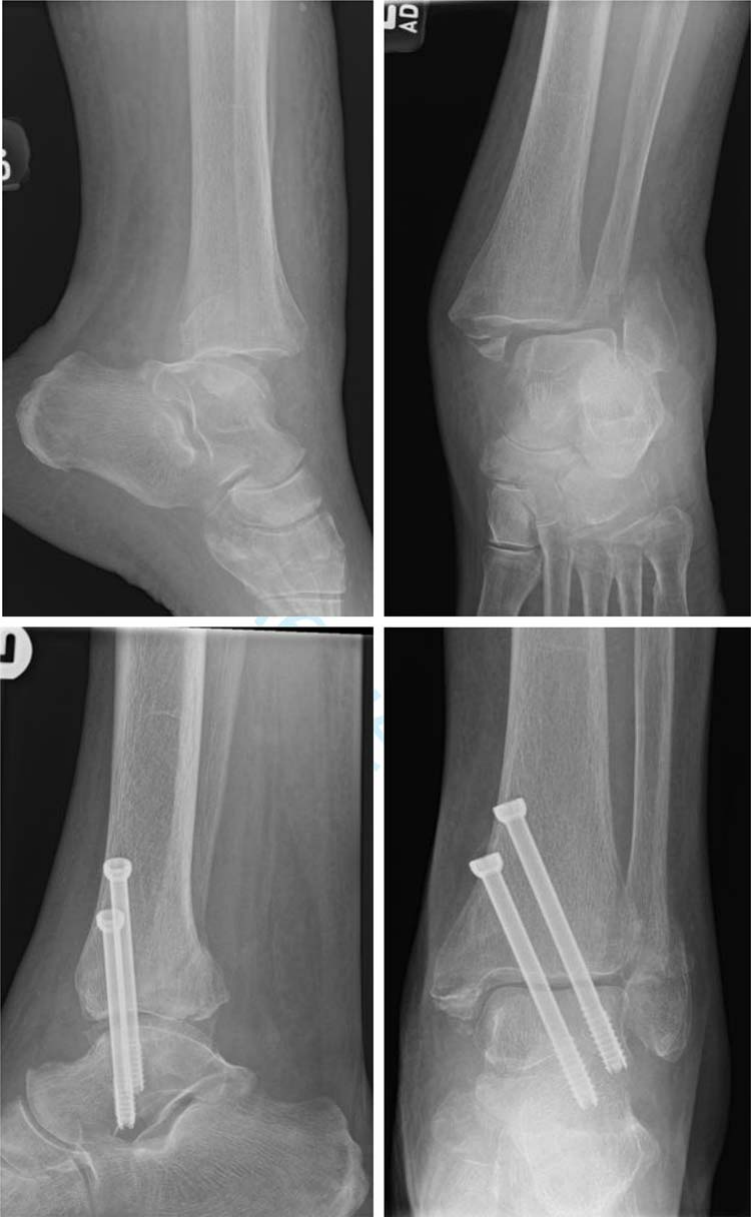
Top—Fracture dislocated bimalleolar ankle fracture. Bottom—6 weeks post-operatively showing maintained tibiotalar alignment.

Overall, four out of six patients (66.7%) had satisfactory post-op radiographs with no evidence of talar shift, non/mal-union, widening of the syndesmosis or screw loosening (Figs 1 and 2).

**Figure 3:**
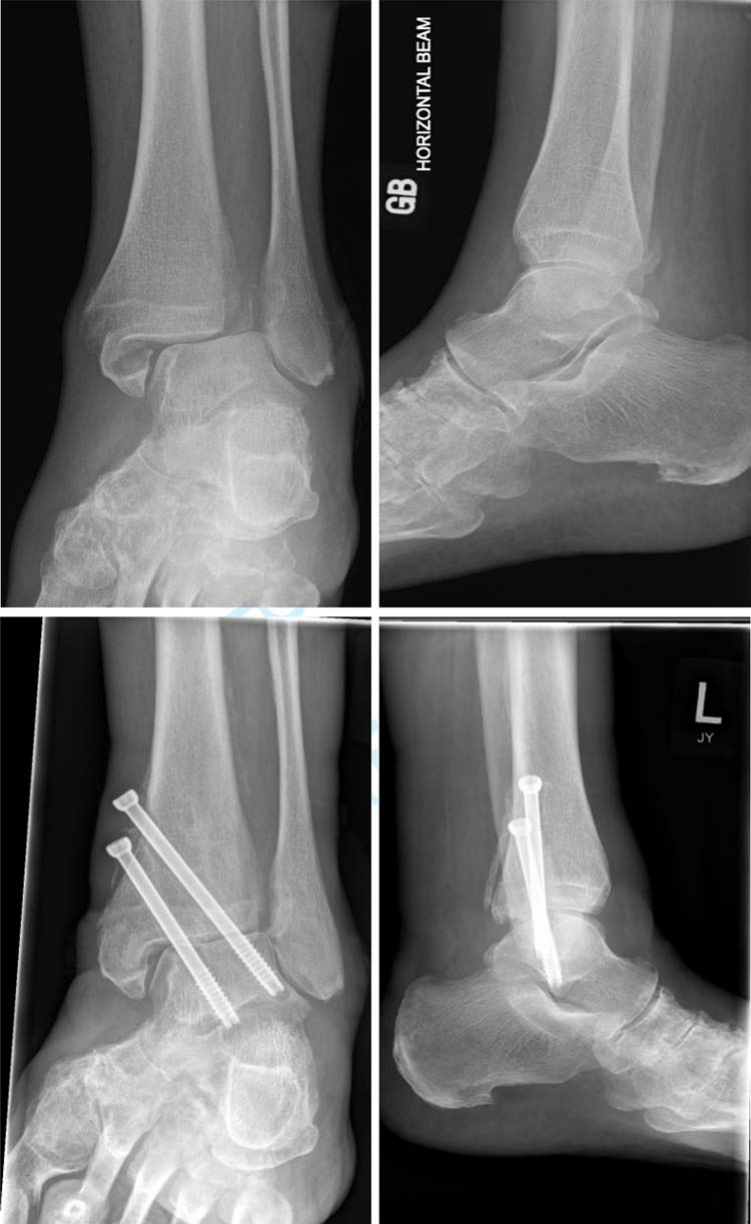
Top—Bimalleolar ankle fracture with talar shift. Bottom—Radiograph at 5 months showing small amount of talar shift.

One patient (16.7%) with a bimalleolar (posterior and medial malleoli involvement) fracture had evidence of minor talar shift at 5 months, but no other significant radiological findings. Additionally, the patient reported no pain, and mobility recovered to a preoperative level (Fig. 3).

One patient (16.7%) had surgery at 2 weeks post-injury. Complete reduction of the talus was not achievable closed, and poor skin condition precluded any open intervention. The ankle was held with two cannulated screws, but screw loosening, widening of the syndesmosis and medial rotation and shift of the talus developed at 6 weeks. However, the patient was at preoperative mobility levels (Frame) and had minimal pain (1 out of 10 severity, relieved by simple analgesia).

### Post-operative pain and mobility

Five out of six (83.3%) patients reported a numerical pain score of zero out of ten at a mean follow-up duration of 10.25 months. One patient (14.3%) reported a one out of ten pain score, which was resolved on taking simple analgesia.

Overall, all patients were able to ambulate, with the use of aids, and weight-bear at a mean follow-up of 10.25 months. Post-operative mobility was unchanged in four of the six patients (66.7%), whereas two patients (33.3%) had a decrease in mobility. One patient was previously independent, but subsequently required a wheeled frame to mobilise at 2 months follow-up. Furthermore, a patient who previously mobilised with a wheeled frame, required a wheelchair, but could weight-bear for a few steps at 14 months follow-up. Both of these patients had deteriorated medically throughout the follow-up period, and the cause of their decreased mobility was likely multifactorial and not solely due to their ankle fracture. Both patients with decreased mobility had zero pain.

### Complications

One patient (14.3%) with a Bimalleolar Weber B fracture was complicated by screws backing out. Nonetheless, radiographs at 3 months showed good talus position and evidence of union. Screws were removed at 3 months, with no further complications. The patient remained pain free with mobility at a preoperative level.

There were no cases of screw breakage. Two patients had evidence of ulceration before fixation, and improvement was observed in one of these patients post-operatively. No patients developed new ulceration post-fixation.

## DISCUSSION

This case-series shows that dual percutaneous tibiotalar cannulated screws can be used to treat unstable ankle fractures in a frail, low demand and elderly patient group. It is a less invasive alternative to the hind foot nail with short operative times. Furthermore, it does not compromise both the tibiotalar and subtalar joints.

The surgical technique aims to ensure that the talus remains under the tibia ensuring the patient has a stable base to mobilise and can mobilise full weight bearing immediately after surgery. The two screws work as internal fixation to stabilise the ankle whilst the fractures unite and are supplemented with a plaster cast for 6 weeks. In our cohort, all the patients could weight bear post-operatively. The ability to weight-bear is essential to elderly patients as it reduces the poor health outcomes associated with being non-ambulatory and reduces their care requirements [7].

A further aspect of the surgical technique is that the approach for the insertion of two screws is quick and minimally invasive, with just two percutaneous incisions. This ensures that damage to tissues is minimal and reduces the risk of wound healing problems, none of which were seen in this study. Similarly, using a minimally invasive technique is likely to reduce the severity of post-operative pain and help patients mobilise earlier. This was seen in our cohort of patients with 83.3% reporting no pain at a mean follow-up of 10.25 months. Additionally, the elderly population are at increased risk from anaesthesia, due to co-morbidities and physiological ageing [8]. One of the benefits of this novel procedure is that the average operation duration is only 29 min. Therefore, the duration of anaesthesia is short, which limits the systemic risk to each patient [8].

### LIMITATIONS

One of the limitations of this study is the small sample size which limits the generalisability of the study. A further limitation is the length of the follow-up period, with mean follow-up of 10.25 months. This short follow-up fails to demonstrate how the cohort progressed over time and whether they suffered long-term sequalae from their injury and fixation technique.

## CONCLUSIONS

This study provides clinical data showing that dual percutaneous tibiotalar cannulated screws can be used to treat unstable ankle fractures in the low demand elderly patient. Short-term follow-up shows that 83.3% of patients were pain free post-operatively, and the majority maintained their preoperative level of mobility.
